# Are we reluctant to talk about cultural determinants?

**Published:** 2011-04

**Authors:** Sanjay Chaturvedi, Narendra K. Arora, Rajib Dasgupta, Ashok K. Patwari

**Affiliations:** Department of Community Medicine, University College of Medical Sciences, Delhi, India; * The INCLEN Trust International, New Delhi, India; ** Centre of Social Medicine & Community Health, Jawaharlal Nehru University, New Delhi, India; + International Health, Center for Global Health & Development, School of Public Health Boston University, Boston, MA, USA

Epidemiologists and public health managers generally harbour a belief that cultural determinants of health would be suitably taken care of if the broader domain of social causations is addressed. Is this placing the cart before the horse? Several of the social determinants may be mere proxies of deeper, and probably causal, cultural determinants. The recently published report of the WHO Commission on Social Determinants of Health is also short of expectations on this score^1^. It is customary to deal with income, education and occupation while remaining oblivious to the fact that some of the cleanest people of the world are from the most deprived tribal areas, and several communities could achieve what they did, not because of their per capita income but for their deeply rooted cultural strengths. In contrast, community based systematic resistance to supplementary immunization activities of polio eradication campaign witnessed in several pockets in India, Pakistan, Afghanistan and Nigeria cannot be totally explained as behaviour of economically disadvantaged communities^2^,^3^. There has been little recognition of the component of cultural resistance behind this phenomenon, and sometimes the imperatives of political correctness smothers such enquiries. On another related front, while privatization of health care is being strongly advocated, nearly all of the sex-selective abortions in South Asia are being conducted and abated by the private sector – and some of the richest and educated people in this part of the world are involved, as clients or as providers. Can this phenomenon be deconstructed without understanding the cultural determinants that sustain male child preference, cynically goaded by dowry system in marriage? Can we possibly explain the continued marginalization of natives, aborigines and tribal people across the globe without analysing the cultural attributes of dominant occupants of the land?^4^–^5^ The time has come for a dedicated identification of such determinants of health instead of covering them up with proxy social factors.

The influence of culture is evident on how people eat, work, conduct themselves, seek care, and react to health promoting/compromising messages. Culture also shapes their constructs on diagnostic categories and syndromes. It also determines their sexuality. To the epidemiologist, the question is - are our existing research methods good enough to capture this influence? It would be difficult to evolve such methods unless we are able to harness and integrate the powers of epidemiology, anthropology, and other related disciplines. In this context, it is relevant to revisit a fact that quantitative epidemiology and qualitative methods are often acknowledged as complementary but rarely integrated. The emerging concept of cultural epidemiology is an attempt in this direction as the EMIC (Explanatory Model Interview Catalogue) framework has offered some partial solutions^6^,^7^. However, one needs to go beyond EMIC tools, which, to a large extent, end up addressing the ‘measurement’ domain alone while the other two - ‘causal thinking’ and ‘programme design’ also need to be addressed within the same framework.

At the ideological level, there are certain unresolved issues between epidemiologists and behavioural scientists. Sociologists denounce medicalisation of public health while epidemiologists consider the culture to be merely a confounding in causal models^8^. The basic premises of cultural epidemiology are based on a broad agreement with qualitative methods. Cultural epidemiology argues against reducing human cases to sets of variables. Recovery of ‘missing persons’ under the mass of variables is also the aim of qualitative approach. However, within the realm of qualitative enquiry, there are some serious issues like ‘etic’ versus ‘emic’. While the ‘etic’ represents the investigators’ perspective - also referred as ‘sensitizing concepts’^9^, the ‘emic’ offers the participants’ perspective. Often in this debate, one tends to lose sight of the fact that researcher also harbours his/her own ‘emic’. Culture is the context in which the research is performed and inferences are co-constructed. It shapes thoughts and actions of both: the subjects as well the investigators. Researchers may not necessarily be aware of the influences of dominant cultural ideology on the way they conduct, interpret, disseminate, and use their research. Objectivity is not value neutral, and, therefore, needs to be redefined to factor the biases associated with dominant cultural ideology and the contemporary market of knowledge. At the very least, researchers need to acknowledge a subtle conflict of interest^10^.

Human societies everywhere have had cultural constructs woven around pregnancy, birth, child rearing, marriage, sex and death. Many of these continue to influence the communities even after acculturation and rural-urban or transcontinental migration. Religion has also a great influence of these practices and may create remarkable inter-group differences within a same regional or ethnic identity. With their merits and demerits, these have a significant bearing on reproductive and child health and need to be factored while designing health programmes that facilitate integration of traditional and modern practices. While the list of health compromising practices is very long and this may be because of a particular orientation and disease-centric nature of enquiry and evidence-base, the good practices are also sizeable in number and demand serious documentation. Between these two distinct categories, there is a gray zone of cultural practices where one can hardly exercise any measure of certitude. Several of the apparently harmful practices may also be a manifestation of long standing deprivations and non-availability of health services - and mark a transition from reasoned choice/compromise to the realm of cultural wisdom.

Use and abuse of a wide range of tobacco products and alcohol at the scale is also goaded by the level of cultural acceptability these products enjoy in the public space. This may vary across different sections of society and gender. In several regions, there is virtually no resistance to tobacco in spite of awareness about its harmful effects. Societal acceptability for alcohol abuse is a huge global problem, and its health outcomes are of pandemic proportions^11^. In developing countries, maternal and child health pays a very heavy price for habitual alcohol abuse by their men^12^,^13^. Already meagre resources are depleted, households gradually fall in a debt trap, men die earlier, children are left to mothers and there is no one to care for the mother. In several regions, men’s alcoholism is perhaps the most important yet under-represented cause of chronic undernutrition among women and children. To an extent, this is happening in developed parts of the world as well^14^.

Gender violence; violence against children, weak, poor and voiceless people; violence amongst youth; and road rage are some of the phenomena that cannot be sustained unless supported by some degree of cultural acquiescence. Several societies continue to associate violence with youth, adventure and masculinity; events of domestic violence, and even rape are not found worthy of reporting; and inexplicable mass violence is systematically rationalised by many through ideological constructs is essentially cultural and needs to be examined in that light^15^,^16^.

In today’s world, the market is weaving its own culture while using almost all the channels of communication to mould and monitor the meanings which people derive and experience for anything from product to policy. Driven largely by profit, this can initiate a process of mass disempowerment through misinformed decision making. Health education and social mobilization campaigns sometimes face stiff opposition from cultural beliefs of target audience but the same people look increasingly vulnerable to market forces much against their cultural diktats. The drivers of market can hardly be expected to stimulate awareness which examines the ethic of consumption that bargains with people’s health for private profit. Health promotion campaigns generally spread messages of moderation, caution and thoughtful restraint; often at odds with the market’s maxim of higher consumption of a range of health compromising products and ideas through attractive role models and lifestyle appeals. Though the health messages urge people to ‘just say no’ to hazardous choices, at the end of the day these might as well be reared to ‘go for it’ through a relentless mass counselling by hidden and not so hidden persuaders. This is chiefly visible in: unhealthy food culture; unhealthy beauty-culture that is inherently Eurocentric and subtly racist in its construct; excessive portrayal of gender identity and sexuality; risky behaviours, especially amongst youth; substance abuse; easily avoidable filth and noise generating behaviours^17^–^19^.

Biomedical thinking has increasingly adopted a narrow ‘causal pragmatism’^20^. In such a proximal-distal divide, cultural determinants can only be relegated to the distal, increasing the ‘causal distance’- notwithstanding the probability that in many instances the ‘distal’ causes may indeed be decisive. Culture may hold explanatory powers of variable strengths in different situations, but can also be an attractive paradigm to epidemiologists in their quest for unifying explanations for seemingly diverse situations. The logic that cultural factors are part of the wider gamut of social determinants may prove to be specious. The contemporary paradigm of social determinants is inadequately equipped to deal with a seemingly more complex web of cultural causations. We may need a dedicated space to examine and intervene in this rather amorphous area of cultural epidemiology.
